# Peptide‐Functionalized Fluorescent Particles for In Situ Detection of Nitric Oxide via Peroxynitrite‐Mediated Nitration

**DOI:** 10.1002/adhm.201700383

**Published:** 2017-05-17

**Authors:** Jason Y. H. Chang, Lesley W. Chow, W. Michael Dismuke, C. Ross Ethier, Molly M. Stevens, W. Daniel Stamer, Darryl R. Overby

**Affiliations:** ^1^ Department of Bioengineering Imperial College London London SW7 2AZ UK; ^2^ Department of Ophthalmology Duke University School of Medicine Durham NC 27710 USA; ^3^ Department of Materials, Department of Bioengineering, and Institute of Biomedical Engineering Imperial College London London SW7 2AZ UK; ^4^ Wallace H. Coulter Department of Biomedical Engineering Georgia Institute of Technology and Emory University Atlanta GA 30332 USA

**Keywords:** endothelial cells, immunoassays, nitric oxide detection, peptide biosensors, peroxynitrite

## Abstract

Nitric oxide (NO) is a free radical signaling molecule that plays a crucial role in modulating physiological homeostasis across multiple biological systems. NO dysregulation is linked to the pathogenesis of multiple diseases; therefore, its quantification is important for understanding pathophysiological processes. The detection of NO is challenging, typically limited by its reactive nature and short half‐life. Additionally, the presence of interfering analytes and accessibility to biological fluids in the native tissues make the measurement technically challenging and often unreliable. Here, a bio‐inspired peptide‐based NO sensor is developed, which detects NO‐derived oxidants, predominately peroxynitrite‐mediated nitration of tyrosine residues. It is demonstrated that these peptide‐based NO sensors can detect peroxynitrite‐mediated nitration in response to physiological shear stress by endothelial cells in vitro. Using the peptide‐conjugated fluorescent particle immunoassay, peroxynitrite‐mediated nitration activity with a detection limit of ≈100 × 10^−9^ m is detected. This study envisions that the NO detection platform can be applied to a multitude of applications including monitoring of NO activity in healthy and diseased tissues, localized detection of NO production of specific cells, and cell‐based/therapeutic screening of peroxynitrite levels to monitor pronitroxidative stress in biological samples.

## 1 Introduction

Nitric oxide (NO) is a diatomic free radical with important physiological roles across multiple biological systems. NO rapidly diffuses across cell membranes and between cells, where it acts as a signaling molecule to modulate vascular homeostasis,^1^, ^2^, ^3^, ^4^, ^5^ neuronal activity,^6^, ^7^ and immunological processes.^8^ NO dysregulation has been linked to the pathogenesis of Parkinson's and Alzheimer's disease,^9^, ^10^, ^11^ cardiovascular disease,^12^, ^13^, ^14^, ^15^ glaucoma,^16^, ^17^, ^18^, ^19^, ^20^ and cancer.^21^, ^22^ The detection and quantification of NO is therefore important for understanding physiology and pathophysiology of disease‐relevant tissues, but the reactive nature of NO and its typically short half‐life (on the order of seconds^1^, ^2^, ^4^, ^6^, ^8^, ^23^) make accurate measurement of NO challenging.

To measure NO within biological systems, researchers generally rely on indirect detection methods to assess NO‐derived products, such as nitrates, nitrites, or post‐translational modifications of proteins that form nitrosothiols or 3‐nitrotyrosines. The Griess assay is one of the most widely used NO detection techniques that measures the concentration of nitrite (NO_2_^−^) produced in biological fluids after the oxidation of NO. Although the Griess assay provides a useful indication for NO production with a detection limit of 0.5–1 × 10^−6^ m,^23^, ^24^, ^25^, ^26^ the assay is unable to resolve low nanomolar concentrations typical of NO‐mediated signal transduction. For instance, the activation of soluble guanylate cyclase that produces cyclic guanosine monophosphate responsible for maintaining vascular tone requires a minimum NO concentration of 5–10 × 10^−9^ m, with typical physiological NO concentrations on the order of hundreds of nanomoles (100–500 × 10^−9^ m)^9^, ^10^, ^23^, ^27^, ^28^ that are typically below the detection limit of the Griess assay. Other factors such as the type of buffer or the presence of amino acids can interfere with the Griess reaction.^29^, ^30^ Fluorescent probes^31^, ^32^, ^33^, ^34^ and electrochemical detection methods^35^, ^36^ have also been used for real‐time detection of NO produced by cells in culture. However, fluorescent probes have been shown to interact nonspecifically with other reactive oxygen and nitrogen species (ROS/RNS)^37^ and have reduced sensitivity to NO (from the nanomolar to the micromolar range) in biological samples when compared to cell‐free conditions (Table S1, Supporting Information).^31^, ^38^, ^39^, ^40^ Furthermore, measurement of NO within native tissues is technically challenging due to limitations with accessibility and extraction of samples. Hence, there is a need for an NO biosensor that can resolve nanomolar concentrations of NO while providing spatiotemporal information within biological systems in situ.

3‐Nitrotyrosine has been identified as a “footprint” of NO‐dependent nitroxidative stress.^24^, ^41^, ^42^ The process of tyrosine nitration is an oxidative post‐translational modification, driven by NO‐derived oxidants, such as peroxynitrite (ONOO^−^; ONOOH) and nitrogen dioxide radical (^•^NO_2_), that yield 3‐nitrotyrosine. Under normal physiological conditions, low levels of 3‐nitrotyrosine can be detected in healthy tissues, which reflect basal steady‐state levels of nitration and oxidation that occur in vivo. However, nitration end products increase several‐fold once the formation of oxidants and NO is augmented (e.g., during inflammation).^41^ This results in elevated levels of 3‐nitrotyrosine, which is commonly associated with progression of multiple diseases in both human and animal models.^9^, ^13^, ^21^, ^22^, ^43^, ^44^

Nitration of tyrosine residues is predominately mediated through the peroxynitrite‐dependent pathway, which incorporates a nitro (—NO_2_) group to the aromatic ring to form 3‐nitrotyrosine.^45^ The formation of peroxynitrite is generated through a diffusion‐limited reaction between NO and superoxide (O_2_^•−^) radicals. Under physiological conditions, both peroxynitrite anion (ONOO^−^) and its protonated form peroxynitrous acid (ONOOH) are present in biological systems and can participate in the oxidation of biomolecules. Peroxynitrite is a short‐lived oxidant species that readily reacts with carbon dioxide (CO_2_) to yield an intermediate adduct, nitrosoperoxocarboxylate (ONOOCO_2_^−^), which quickly homolyzes into carbonate (CO_3_^•−^) and ^•^NO_2_ radicals. Alternatively, peroxynitrous acid (ONOOH) can undergo a similar homolytic fission to generate hydroxyl (^•^OH) and ^•^NO_2_ radicals. These one‐electron oxidants promote the formation of a tyrosyl radical intermediate (Tyr^•^), which then combines, at diffusion‐limited rates, with ^•^NO_2_ to yield 3‐nitrotyrosine (as illustrated in Figure S1 of the Supporting Information).^23^, ^24^, ^41^, ^42^, ^45^, ^46^, ^47^, ^48^, ^49^, ^50^ Nitration of tyrosine residues can therefore serve as an indirect indicator for local NO levels. However, conversion of tyrosine to 3‐nitrotyrosine under nitroxidative conditions is very selective, sensitive to local amino acid sequence and selective for specific proteins in vivo.^13^, ^51^, ^52^, ^53^, ^54^ Therefore, identifying nitration‐prone proteins and exploiting their specific amino acid sequences allow us to assess local changes in NO levels.

**Figure 1:**
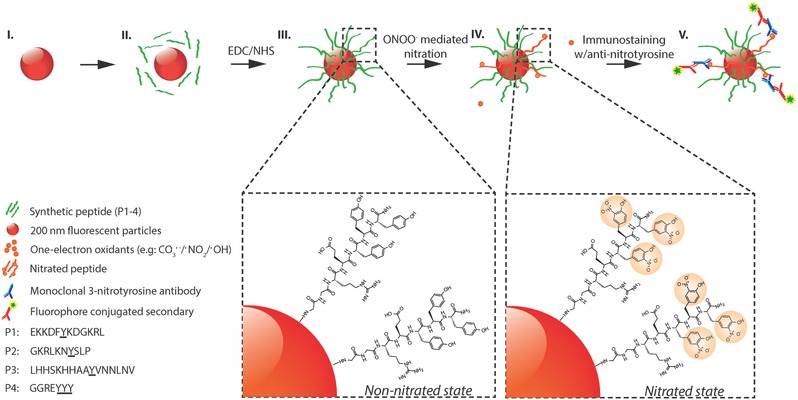
Schematic representation of peroxynitrite‐induced nitration. (I) The fluorescent particle, (II) conjugation of peptides with EDC/NHS cross‐linker (P1–P4), (III) non‐nitrated peptides conjugated to surface of fluorescent particles, (IV) nitration of tyrosine through peroxynitrite‐mediated pathway, (V) immunostaining of nitrated peptides with anti‐nitrotyrosine IgGs and fluorescent secondary IgGs. (Steps I–III): Carboxyl‐functionalized red fluorescent particles (≈200 nm in size) are coated with tyrosine‐containing peptides (P1–P4, green strands). Step IV: Peroxynitrite‐mediated nitration of tyrosine residues resulting in the formation of 3‐nitrotyrosine. Step V: Immunostaining of nitrated peptides with monoclonal anti‐nitrotyrosine IgGs (MAB5404; Millipore) and fluorophore‐conjugated secondary IgGs.

Several site‐specific tyrosine residues from native proteins have been identified as preferential targets for nitration in vivo.^23^, ^44^ Multiple mechanistic studies have shown that the local primary structure of peptides plays a crucial role in determining site‐specific nitration of tyrosine residues, whereby proximal charged residues increase nitration yield while hydrophobic residues tend to yield lower nitration through possible steric hindrance of bulky side groups.^12^, ^49^, ^55^, ^56^, ^57^, ^58^, ^59^, ^60^ Therefore, by mimicking the amino acid sequence of nitration‐prone sites within native proteins, we have the opportunity to design and synthesize peptide‐based biosensors that are sensitive to local changes in NO production. In this study, we synthesized four different tyrosine‐containing peptides (P1–P4) and compared their relative sensitivity and specificity toward various ROS and RNS. Three of the peptides were derived from nitration‐prone proteins, with two of these peptides (P1–P2) from prostacyclin synthase (PGI_2_ synthase)^13^, ^53^, ^60^, ^61^, ^62^ and one peptide (P3) from manganese superoxide dismutase (MnSOD).^12^, ^47^, ^49^, ^51^ Additionally, we designed the fourth peptide (P4) with multiple tyrosines flanked by charged amino acids with the aim to amplify nitration. Incorporating adjacent charged amino acids such as glutamate (E) and arginine (R) has been shown to enhance the selective nitration of tyrosine residues in proteins.^49^, ^52^, ^58^, ^60^ As illustrated in **Figure** **1**, each of the synthetic peptides was covalently bound to fluorescent particles (FPs) such that the nitration reaction was localized to the FP surface. This enables us to deliver and track these peptide–FPs in specific biological tissues of interest and detect changes in local NO production, without disturbing the normal tissue architecture, function or losing spatiotemporal information. In the presence of NO‐derived oxidants, the tyrosine residues within the peptide become nitrated resulting in the formation of 3‐nitrotyrosine. The detection of 3‐nitrotyrosine residues was carried out by incubating the peptide–FPs with a commercially available monoclonal antibody selective for 3‐nitrotyrosine followed by incubation with a secondary fluorescently labeled antibody. The readout was assessed based on the immunofluorescence intensity of 3‐nitrotyrosine normalized by the fluorescence intensity of the FPs themselves to control for possible variations in the number of particles. The goal of this study was to demonstrate a proof‐of‐concept that our peptide–FP biosensors are capable of detecting peroxynitrite‐mediated nitration in vitro at low NO concentrations as well as detecting NO released from endothelial cells in response to physiological levels of shear stress. We also compare our peptide–FP biosensors against the standard Griess assay and the fluorometric methods to measure NO.

## 2 Results and Discussion

### 2.1 Sensitivity and Specificity of Unbound Peptides to Nitration

**Figure 2:**
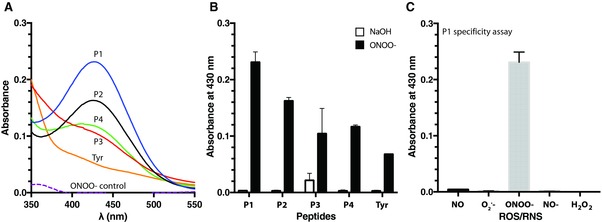
3‐Nitrotyrosine detection with UV–vis spectrophotometry. A) Representative spectra of 3‐nitrotyrosine detection for each peptide. Peptides (P1–P4; 1 × 10^−3^ m) and l‐tyrosine (Tyr; 1 × 10^−3^ m) were exposed to peroxynitrite (0.5 × 10^−3^ m) in phosphate buffered saline (pH 7.4) for 1 h at 37 °C; nitration yields were determined with UV–vis. The presence of 3‐nitrotyrosine in P1 (solid blue line), P2 (solid black line), P3 (solid red line), P4 (solid green line), and Tyr (solid orange line) was shown as an increase in absorbance at 430 nm, where it was compared to peroxynitrite alone (ONOO^−^; dashed purple line). B) Average 3‐nitrotyrosine signal for peroxynitrite‐treated peptides (black bars; *N* = 3) compared to vehicle‐treated control peptides (white bars; *N* = 3). 3‐Nitrotyrosine yields were measured at 430 nm using UV–vis spectrophotometry. C) Representative peptide (P1) specificity assay treated with various reactive oxygen and nitrogen species (ROS/RNS; 0.5 × 10^−3^ m). Absorbance values detected at 430 nm (*N* = 3). Vehicle control = 0.3 m NaOH. Error bars represent SD.

To determine the sensitivity of unbound peptides to nitration, each peptide was solubilized at the same molar concentration (1 × 10^−3^ m) and treated with saturating levels of peroxynitrite (0.5 × 10^−3^ m) in phosphate buffered saline (PBS) for 1 h at 37 °C. Sodium hydroxide (0.3 m NaOH) was used as a vehicle control, as peroxynitrite is supplied in NaOH to maintain its stability. 3‐Nitrotyrosine yields were measured at 430 nm using UV–vis spectrophotometry (**Figure** **2**). P1 (EKKDFYKDGKRL; derived from PGI_2_ synthase) was the most susceptible to peroxynitrite‐mediated nitration, exhibiting a 77‐fold increase in 3‐nitrotyrosine signal compared to vehicle‐treated control (0.231 ± 0.018 vs 0.003 ± 0.001; *N* = 3; *p* < 10^−5^). P2 (GKRLKNYSLP; also derived from PGI_2_ synthase) showed a 54‐fold increase in 3‐nitrotyrosine signal compared to vehicle‐treated control (0.163 ± 0.006 vs 0.003 ± 0.001; *N* = 3; *p* < 10^−6^). P3 (LHHSKHHAAYVNNLNV; derived from MnSOD) displayed a high background signal (see Figure S2 of the Supporting Information) and exhibited only a fivefold increase in 3‐nitrotyrosine signal compared to vehicle‐treated control (0.104 ± 0.045 vs 0.022 ± 0.012; *N* = 3; *p* = 0.036). P4 (GGREYYY) containing three tyrosines yielded a 39‐fold increase in 3‐nitrotyrosine signal compared to vehicle‐treated control (0.117 ± 0.003 vs 0.003 ± 0.002; *N* = 3; *p* < 10^−6^). l‐tyrosine (Tyr) at 1 × 10^−3^ m was used to determine whether local amino acid sequence surrounding the tyrosine residue influences nitration. Tyr alone yielded a 23‐fold increase in 3‐nitrotyrosine signal compared to vehicle‐treated control (0.068 ± 0.001 vs 0.003 ± 0.001; *N* = 3; *p* < 10^−6^), which was smaller than the relative change measured for P1, P2, and P4. These data demonstrate that the flanking amino acid sequence influences tyrosine nitration and that peptides derived from nitration‐prone proteins tend to be more sensitive to peroxynitrite compared to free tyrosine. When comparing the biomimetic peptides (P1–P4) derived from nitration‐prone proteins against l‐tyrosine alone, it was clear that the surrounding amino acids help modulate the site‐specific tyrosine nitration, as reflected in native proteins.

We also examined the dose‐dependent response of each peptide over a range of peroxynitrite concentrations (10–500 × 10^−6^ m) to determine their relative detection limits (Figure S2, Supporting Information). Each peptide produced different levels of 3‐nitrotyrosine signal in response to increasing levels of peroxynitrite, further suggesting that nitration is a selective process that is sensitive toward the local amino acid sequence. Furthermore, we examined the specificity of each peptide sequence toward other ROS/RNS, including NO, NO**^−^**, O_2_ **^•−^**, and H_2_O_2_. In comparison to the increased 3‐nitrotyrosine signal observed for P1 following treatment with peroxynitrite, there was negligible signal in response to the other ROS/RNS (Figure 2C). These data demonstrate that peroxynitrite is the key intermediate leading to tyrosine nitration. Similar results were observed for the other peptide sequences (Figure S3, Supporting Information).

Peptides P1 and P2, derived from PGI_2_ synthase, were most susceptible toward peroxynitrite‐mediated nitration. The amino acid sequence of P1 consists of alternating acidic (E and D) and basic (R and K) residues in close proximity to the hydrophobic residues (F and Y) while P2 consists of mainly basic (R and K) and polar (N and S) residues adjacent to the target tyrosine. This may have created a local hydrophilic environment around the tyrosine residue, increasing the exposure and susceptibility of tyrosine residues to peroxynitrite‐meditated nitration. In contrast, P3, derived from MnSOD, consists of several hydrophobic residues (H, A, and V) that may limit the accessibility of peroxynitrite to the target tyrosine residue, thus resulting in lower 3‐nitrotyrosine yield. P4 was designed with an acidic and basic residue proximal to three tyrosine residues to potentially amplify the tyrosine nitration signal. Interestingly, the ratio of nitrated peptide to vehicle‐treated peptides was greater in P1 and P2 when compared to P4, suggesting that the nitration process is highly selective and sensitive to the amino acid sequence, and the presence of additional tyrosines does not necessarily yield greater nitration. In addition to local amino acid sequence, the nitration of tyrosine residues within native proteins is also influenced by the existence of secondary and tertiary structures. Furthermore, the interaction between peroxynitrite and metal‐ or heme‐prosthetic group binding sites^44^ has shown to promote the formation of secondary radicals that enhances peroxynitrite‐mediated nitration. Therefore, the probable lack of secondary and tertiary structures and transition metals in our peptide biosensors could lead to lower nitration yields, which may partially explain the results shown with P3. Furthermore, the detection limit for peroxynitrite‐mediated nitration with UV–vis was ≈10 × 10^−6^ m, which is not sensitive enough to measure basal physiological concentrations around 100 × 10^−9^ m.^23^, ^24^ Therefore, to overcome this detection limit we incorporated immunochemical techniques for specific 3‐nitrotyrosine labeling in conjunction with our peptide sensors to amplify the detection of nitrated peptides.

### 2.2 Characterization of Peptide–FPs to Nitration

**Figure 3:**
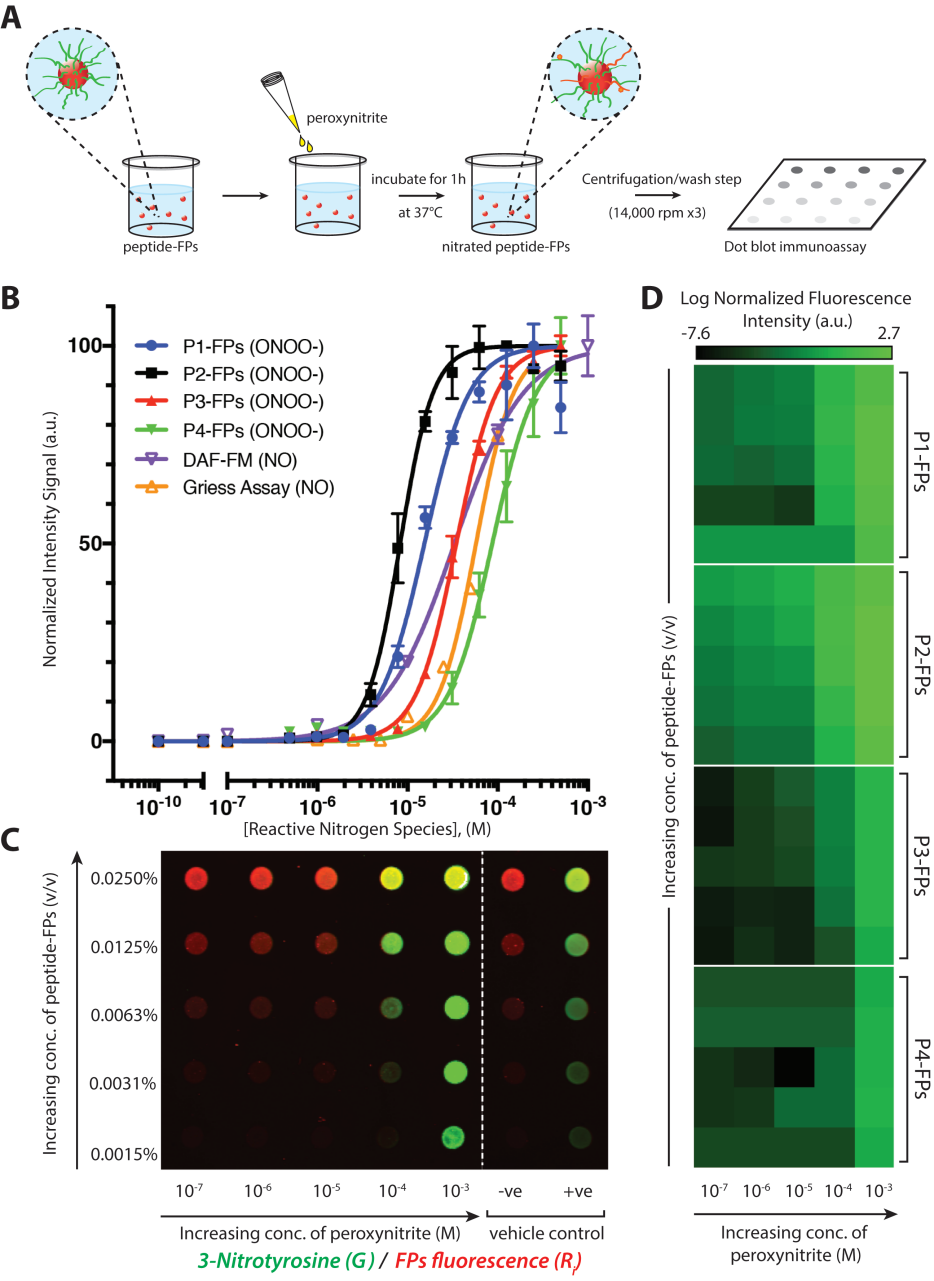
Representative immunoassay of peroxynitrite‐induced nitration of fluorescent particle complexes. A) Schematic representation of peptide–FP complexes treated with peroxynitrite in a 96‐well plate and incubated at 37 °C for 1 h, followed by centrifugation/wash step (at 14,000 rpm for 10 min, for three times) and dot blotted onto a nitrocellulose membrane. B) Comparison of normalized dose–response curves of each peptide–FP complex against the DAF‐FM probe and Griess assay as a function of increasing concentration of reactive nitrogen species (either NO or ONOO^−^). Peptide–FPs were treated with peroxynitrite (500 × 10^−9^ m to 500 × 10^−6^ m), while the DAF‐FM and Griess assay were used to detect NO/NO_2_^−^. Peptide–FPs were loaded at 0.01% (v/v) concentration (*N* = 2). C) Representative immunoarray of 3‐nitrotyrosine detection sensitivity as a function of concentration of peptide–FPs or peroxynitrite (representative immunoarray of P1‐FPs). Fluorescent particles are shown in red; anti‐nitrotyrosine immunofluorescence signal is shown in green; vehicle‐treated controls: [sodium hydroxide (0.3 m NaOH; −ve); 3‐nitrotyrosine‐conjugated fluorescent particles (+ve)]. Fluorescence was detected with a two‐channel infra‐red scanner (Odyssey; Licor). D) Averaged fluorescence intensity of 3‐nitrotyrosine detection as a function of peptide–FPs concentration (*y*‐axis; same concentrations as panel C) or peroxynitrite concentration (*x*‐axis) presented in a heat map (*N* = 3). Each dot blot fluorescence signal was normalized against the particle's autofluorescence to account for variations of fluorescent particle concentration. Normalized fluorescence intensity is shown on a log‐scale to show the sensitivity of the 3‐nitrotyrosine antibody signal.

We conjugated the peptides to FPs (200 nm diameter) to confine and concentrate the peptide sensors for immunochemical detection (**Figure** **3**A). 3‐Nitrotyrosine signal was identified using fluorescently labeled immunoglobulin G (IgG) in a dot blot immunoassay. The sensitivity of each peptide–FP complex toward peroxynitrite‐mediated nitration was determined by generating dose–response curves utilizing 0.01% (v/v) peptide–FP solutions treated with increasing concentrations of peroxynitrite (500 × 10^−9^ m to 500 × 10^−6^ m; Figure 3B; Figure S4 of the Supporting Information). P2–FPs were found to be the most sensitive toward nitration, with an EC_50_ value of 8.3 × 10^−6^ m while P1–FPs, P3–FPs, and P4–FPs had EC_50_ values of 15, 35, and 87 × 10^−6^ m, respectively (**Table** **1**). We also compared the peptide–FPs against standard NO detection methods, including the Griess assay and 4‐amino‐5‐methylamino‐2′,7′‐difluorescein (DAF‐FM) fluorescent probe (1 × 10^−6^ m; Figure 3B; Figure S5, Supporting Information) that showed EC_50_ values of 57 and 33 × 10^−6^ m, respectively, when treated with increasing concentrations of NO donor (peroxynitrite, propylamine propylamine NONOate (PAPA NONOate); 0.1 × 10^−9^ m to 1 × 10^−3^ m; Table 1). The linear range of detection for each peptide–FP complex was also calculated and benchmarked against the Griess assay and DAF‐FM probe (Table 1), revealing that the linear range of detection for P1–FPs and P2–FPs was comparable to Griess but considerably smaller than the linear range covered by DAF‐FM under cell‐free conditions. We then went on to determine whether the sensitivity of each peptide–FP complex toward peroxynitrite depended on peptide–FP concentration (0.0015–0.025% (v/v); *y*‐axis) over a wide range of peroxynitrite concentrations (100 × 10^−9^ m to 1 × 10^−3^ m; *x*‐axis), as shown in Figure 3C.

**Table 1:** Comparison of EC_50_ values as a function of increasing concentration of reactive nitrogen species (RNS). Calibration needs to be performed before each experimental set to determine linear range of detection. CI, confidence interval

Detection method	[RNS]	EC_50_ a)	95% CIa)	*R* ^2^	Linear range of detectiona)
P1–FPs	ONOO^−^	15.4	13.5–17.5	0.981	≈1–30
P2–FPs	ONOO^−^	8.3	7.8–8.9	0.994	≈0.5–15
P3–FPs	ONOO^−^	34.7	33.4–36.0	0.998	≈4–65
P4–FPs	ONOO^−^	87.1	80.5–94.2	0.989	≈8–125
Griess assay	NO_2_^−^	33.2	28.8–38.2	0.996	≈2–100
DAF‐Fm	NO	57.4	55.1–59.9	0.995	≈0.1–100

^a)^ Concentrations represented in micromolar (× 10^−6^ m).

To better illustrate the relative sensitivities of each peptide–FP complex, the averaged 3‐nitrotyrosine signal was presented in a pseudocolor‐heatmap (*N* = 3 per peptide–FP complex), as shown in Figure 3D. From the heatmap, P2–FPs exhibited the most consistent dose‐dependent response. When comparing the detection limits of these peptide–FP complexes, P2–FPs had the greatest sensitivity at the lower ranges of peroxynitrite treatment, achieving detection limits of ≈100 × 10^−9^ m, while P1–FPs showed more variability in their detection of sub‐micromolar levels of peroxynitrite. P3–FPs and P4–FPs were both less sensitive toward peroxynitrite‐mediated nitration, especially at the micromolar range of peroxynitrite. Moreover, both P3–FPs and P4–FPs showed greater variability in their immunofluorescence signals compared to P1–FPs and P2–FPs. These results are consistent with the UV–vis data, showing a similar trend in peptide sensitivity toward peroxynitrite‐mediated nitration, but with further enhanced detection sensitivity by 2 orders of magnitude (from 10 × 10^−6^ m to 100 × 10^−9^ m) for P1–FPs and P2–FPs. Interestingly, our peptide–FP complexes achieved lower detection limits when compared to the Griess assay (≈1 × 10^−6^ m) but did not achieve the detection limits of the DAF‐FM probe (≈10 × 10^−9^ m) under cell‐free conditions. Taken together, this suggests that P1–FPs and P2–FPs are suitable for detecting sub‐micromolar levels of NO‐derived oxidants, affirming the use of immunochemical methods to amplify the 3‐nitrotyrosine signal and improve detection.

One limitation of the peptide‐bound FPs is potential peptide loading differences on the surface of the fluorescent particles, which could occur due to the individual peptide sequences. These potential loading differences could explain, in part, differences in detection sensitivity between peptide–FP complexes. Additionally, the peptides may also interact differently with the surface of the FPs, which could affect the accessibility of peroxynitrite to tyrosine residues and thus its nitration potential. Hence, our platform uses the relative change in 3‐nitrotyrosine signal of each peptide–FP complex.

### 2.3 Detection of Shear‐Mediated Nitric Oxide Produced by Human Umbilical Vein Endothelial Cells (HUVECs)

**Figure 4:**
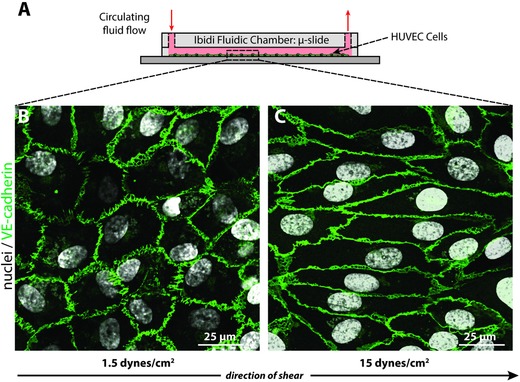
HUVECs exposed to different levels of shear stress. A) HUVECs were sheared under low and high shear stress for 24 h with peptide–FPs circulating in the culture media. B) HUVECs at low shear (1.5 dynes cm^−2^) exhibited the characteristic cobblestone morphology, C) while HUVECs exposed to high shear (15 dynes cm^−2^) showed elongated cell shapes aligned with the direction of flow. Cells were stained for nuclei (DAPI; white) and junctional proteins as an indication of cell monolayer confluency (VE‐cadherin; green), which localized to cell borders. The arrow indicates the direction of laminar flow applied to the cells.

To determine whether our peptide–FPs can detect NO production by living cells, we introduced the peptide–FPs into the circulating media of an in vitro shear stress model whereby the flow chamber is lined with HUVECs exposed to different levels of shear stress over 24 h (**Figure** **4**A). Endothelial cells are shear responsive,^63^, ^64^ exhibiting changes in cell morphology and increased NO production in response to shear stress. HUVECs were exposed to either low (1.5 dynes cm^−2^) or high (15 dynes cm^−2^) physiological levels of shear stress experienced by vascular endothelial cells. Cells exposed to low shear did not align in the direction of flow or exhibit any obvious changes in cell morphology (Figure 4B). In contrast, cells exposed to high shear aligned in the direction of fluid flow after 24 h, changing their morphology from a cobblestone‐like shape to an elongated spindle shape, as demonstrated by immunolabeling for the endothelial cell–cell junctional protein vascular endothelial (VE)‐cadherin (Figure 4C).

**Figure 5:**
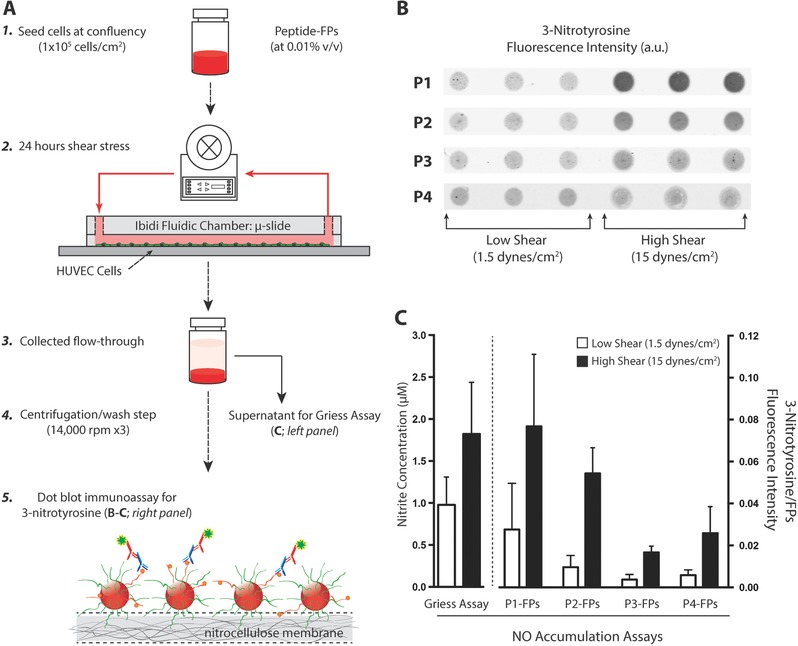
3‐Nitrotyrosine detection of cells under shear stress. A) Schematic representation of HUVECs cultured under low and high shear with the presence of circulating peptide–FP complex to detect NO production. Step 1: Cells were seeded at confluency (1 × 10^5^ cells cm^−2^) and allowed to acclimate overnight (18–24 h). Step 2: Each µ‐slide was loaded with peptide–FP complex (at 0.01% v/v) at the start of the shear experiments. Steps 3–4: After 24 h of shear stress, both media and fluorescent particles were collected and pelleted. Step 5: Peptide–FPs were washed and analyzed for 3‐nitrotyrosine formation (panel B). The collected medium was stored for nitrite detection (by Griess assay). B) Representative dot‐blot immunoassay comparing the amount of 3‐nitrotyrosine binding for each peptide–FP complex (P1–P4) under either low or high shear stress. For each peptide–FP complex, six individual experiments were conducted, each with triplicate measures per experimental condition. C) Comparison of NO accumulation assays. (Left) Griess reagent assay detection for nitrite concentration. Nitrite release (24 h – accumulation) was assayed from the supernatant of each sheared experiment. Data show the averaged nitrite release from HUVECs between low versus high shear (*N* = 22 vs 24; *p* < 0.01). (Right) Averaged 3‐nitrotyrosine signal for each peptide–FP complex normalized against particles autofluorescence to account for loading differences. Data are expressed as mean ± SD for all shear experiments (*N* = 6) except for P1‐FPs and P2‐FPs at low shear with only *N* = 5 each. Error bars represent SD.

To determine the level of NO produced by the cells, the conditioned medium was collected from each individual experiment and subjected to centrifugation. Afterward, the supernatant was collected and used for the traditional Griess reaction assay, while the peptide–FPs at the bottom of the tubes were used for the 3‐nitrotyrosine immunoassay (**Figure** **5**A). The Griess assay served as a positive control for NO detection and a basis for comparison with our NO‐detection platform. Results showed that exposing cells to higher levels of shear stress (15 dynes cm^−2^) resulted in approximately twofold increased levels of nitrite in the media when compared to the low shear condition (1.5 dynes cm^−2^) (1.82 ± 0.62 × 10^−6^ vs 0.98 ± 0.33 × 10^−6^ m; *N* = 24 vs 22; *p* < 0.001) after 24 h (Figure 5C; left panel), consistent with the previous findings.^65^

To compare the relative nitration yields for each peptide–FP complex, the pelleted peptide–FPs were dot‐blotted and analyzed for 3‐nitrotyrosine signals after 24 h of low and high shear stress (Figure 5B). Each of the peptide–FP complexes detected NO production at both low and high levels of shear stress. When comparing the averaged 3‐nitrotyrosine signals (Figure 5C; right panel), P1–FPs had the highest fluorescence signals but also exhibited greater variability in fluorescence signal between experimental samples. P1–FPs showed an approximately threefold increase in shear‐induced nitration when comparing the high shear condition to low shear conditions (*N* = 6 and 5, respectively; *p* < 0.05). P2–FPs had the second highest fluorescence signals while exhibiting less variability between experimental samples. Thus, P2–FPs displayed the largest relative signal in response to increasing shear stress, with an approximately fivefold increase in 3‐nitrotyrosine signal when comparing the high shear condition to low shear condition (*N* = 6 and *N* = 5, respectively; *p* < 10^−5^). These results are consistent with the greater sensitivity of P2–FPs observed in the nitration immunoassay shown in Figure 3. P3–FPs and P4–FPs had lower fluorescence signals but still resolved changes in NO levels between cells exposed to low and high shear stress. For the high shear condition, the average fluorescence intensities increased by approximately fourfold for both P3–FPs and P4–FPs (*N* = 6 for each condition, *p* < 10^−5^ and *p* < 0.05, respectively) when compared to low shear. The relative increase in 3‐nitrotyrosine signal measured by all four peptides (threefold to fivefold) exceeded that measured by Griess assay (twofold) when applied to the same shear‐treated samples. This may be attributable to the low shear baseline concentrations of NO falling below the linear range of detection by the Griess assay at ≈1 × 10^−6^ m (Table 1; Figure 3B). This would tend to decrease the relative change in NO as measured by Griess. In contrast, the linear range of detection, particularly for P2–FPs that extends from 0.5 × 10^−6^ to 15 × 10^−6^ m at 0.01% v/v as used in these studies (Table 1; Figure 3B), would have fully contained the changes in NO levels in response to shear stress. In summary, we have demonstrated that our peptide–FPs detect shear‐induced NO production by HUVECs in culture, through highly selective and specific tyrosine nitration, while also achieving a sensitivity that exceeds the traditional Griess assay.

## 3 Conclusion

This study demonstrates that tyrosine‐containing peptides have the potential to be used as biosensors to detect NO based on tyrosine nitration. We characterized four peptides, three of which were derived from nitration‐prone proteins. By UV–vis, we showed that these peptides had a detection limit of 10 × 10^−6^ m for peroxynitrite, the key intermediate between NO and 3‐nitrotyrosine. This detection limit was improved to 100 × 10^−9^ m by conjugating the peptides to FPs and labeling with fluorescent antibodies against 3‐nitrotyrosine. This exceeded the detection limit of the traditional Griess assay, which is typically 0.5 × 10^−6^–1 × 10^−6^ m. To demonstrate that the peptides are able to detect physiological levels of NO in the presence of endogenous superoxide, we exposed HUVEC cells to laminar shear stress. Peptide‐functionalized FPs contained within the culture media exhibited a threefold to fivefold increase in 3‐nitrotyrosine labeling in response to shear, consistent with shear‐induced NO production that is characteristic of vascular endothelial cells.

NO is a reactive nitrogen species that can proceed down a number of different chemical pathways, including NO_2_^−^/NO_3_^−^ as measured by the Griess assay, lipid nitration, or protein nitration as measured by the current assay. To our knowledge, no single assay measures total NO. A common assumption to all NO‐derived assays is that a relative increase along any one pathway is proportional to the relative increase in total NO production. For example, 3‐nitrotyrosine is formed through the key intermediate, ONOO^−^, which is formed by the reaction between NO and O_2_^**•**−^. Therefore, both NO and O_2_^**•**−^ must be present for these sensors to function properly. Although O_2_^**•**−^ levels are typically kept low by superoxide dismutase (SOD) both in vitro and in vivo, the reaction between NO and O_2_^**•**−^ outcompetes SOD such that ONOO^−^ is produced whenever NO and O_2_^**•**−^ are generated within a few cell diameters of one another.^23^ Data obtained here in the presence of cultured HUVECs demonstrated that conditioned medium contains sufficient O_2_^**•**−^ to detect a shear‐dependent increase in 3‐nitrotyrosine. This likely reflects increased NO production by vascular endothelial cells in response to laminar shear stress,^63^, ^64^ despite laminar shear stress acting to suppress superoxide in other vascular endothelial cell types.^66^ Furthermore, the process of tyrosine nitration produces a stable detectable by‐product,^50^ 3‐nitrotyrosine, which provides a cumulative measure of NO production. This is not unlike the Griess assay or fluorometric readouts that provide a cumulative readout based on NO_2_−/NO_3_− accumulation or irreversible NO binding to ROS/RNS sensitive fluorophores, respectively.

Tyrosine‐containing peptides conjugated to FPs may be useful for detection of NO in vivo. For example, peptide–FPs may be introduced intravenously into animal models to assess NO activity in the general circulation to examine how NO dysregulation is associated with cardiovascular disease in animal models.^12^, ^13^, ^14^, ^15^, ^47^, ^48^ Alternatively, the peptide–FPs may be immobilized within specific tissues and assayed by immunostaining within histological section to detect local NO production in situ that would not be detectible by any existing technique.

## 4 Experimental Section

*Materials and Reagents*: FluoSpheres_580/605_ (carboxylated FPs, 200 nm in diameter) were purchased from Molecular Probes (Invitrogen). 9‐Fluorenylmethoxycarbonyl (Fmoc)‐protected amino acids, Rink amide 4‐methylbenzhydrylamine (MBHA) resin, *N*,*N*‐diisopropylethylamine (DIEA), 2‐(1Hbenzotriazol‐1‐yl)‐1,1,3,3‐tetramethyluronium hexafluorophosphate (HBTU), dichloromethane (DCM), dimethylformamide (DMF), 80:20 dimethylformamide/piperidine premix, and spectroscopic grade acetonitrile (ACN) were purchased from AGTC Bioproducts, UK. PAPA NONOate, Angeli's salt (NO^−^), and 3‐nitrotyrosine were all purchased from Cayman Chemical. The reactive nitrogen species were stored at −80 °C, while 3‐nitrotyrosine was stored at room temperature. l‐tyrosine, hydrogen peroxide (H_2_O_2_) and DAF‐FM were purchased from Sigma. 1‐Ethyl‐3‐(3‐dimethylaminopropyl) carbodiimide hydrochloride (EDC), *N*‐hydroxysuccinimide (NHS), and 2‐(*N*‐morpholino)ethanesulfonic acid (MES) were all obtained from ThermoFisher Scientific. Mouse monoclonal anti‐nitrotyrosine (clone 2A8.2; MAB5404) antibody and xanthine/xanthine oxidase to generate superoxide^70^ were obtained from Merck Millipore; goat anti‐rabbit VE‐Cadherin (XP monoclonal #2500) antibody was obtained from Cell Signaling; Alexa Fluor 488 goat anti‐rabbit IgG secondary antibody was obtained from Life Technologies Inc., and goat anti‐mouse IgG secondary antibody (IRDye 800CW) was obtained from LI‐COR Biosciences.

*Peptide Synthesis*: Four synthetic peptides (P1: EKKDFY_421_KDGKRL–CONH_2_; P2: GKRLKNY_430_SLP–CONH_2_; P3: LHHSKHHAAY_34_VNNLNV–CONH_2_; and P4: GGREYYY–CONH_2_; Figure S3, Supporting Information) were prepared either by manual solid‐phase peptide synthesis (SPPS) or on a peptide synthesizer (Symphony Quartet; Protein Technologies, Inc.) using standard Fmoc SPPS chemistry^67^ on a Rink‐amide MBHA resin. Briefly, Fmoc deprotection was performed with 20% piperidine in DMF for 10 min, repeated twice followed by washes with DMF and DCM. Amino acid couplings were carried out with Fmoc‐protected amino acids (4 equivalents), HBTU (3.75 equivalents), and DIEA (6 equivalents) in DMF for 1–2 h. The peptides were cleaved from the resin and deprotected with 95% trifluoroacetic acid (TFA), 2.5% tri‐isopropylsilane, and 2.5% distilled water (dH_2_O) for 4 h. The TFA was removed using rotary evaporation, and the peptide was precipitated and washed with cold diethyl ether. For purification, the peptide was dissolved in a solution of 4.9% ACN in ultrapure water with either 0.1% TFA or NH_4_OH and purified using reverse‐phase preparative high‐performance liquid chromatography (HPLC; Shimadzu) running a mobile phase gradient of ultrapure water with 5% to 100% ACN containing 0.1% TFA or NH_4_ OH. The Phenomenex C_18_ Gemini column was 150 × 21.2 mm and had a 5 µm pore size and 100 Å particle size. The HPLC fractions were checked for the correct mass using matrix‐assisted laser desorption spectroscopy (MALDI; Waters), and the pure peptide solution was rotary evaporated to remove ACN and lyophilized on a freeze dryer (Labconco). After lyophilization, the purified peptides were confirmed with analytical HPLC and MALDI (Figure S3, Supporting Information) then stored at −20 °C until needed.

*Detection of Nitrated Peptides in Solution*: Peptides were dissolved in PBS (pH 7.4) supplemented with 10% dimethyl sulfoxide at a stock concentration of (10 × 10^−3^ m) then diluted to a working concentration of 1 × 10^−3^ m prior to the nitration experiments. For nitration experiments, each peptide was incubated with various reactive oxygen and nitrogen species (ONOO^−^, NO^−^, NO, H_2_O_2_, and O_2_^•−^; at 0.5 × 10^−3^ m) for 1 h at 37 °C followed by measurements with UV–vis spectrophotometry (Beckman Coulter DU 800). Peroxynitrite was used as an intermediate NO‐derived oxidant to simulate peroxynitrite‐mediated nitration. Peroxynitrite concentration was measured spectrophotometrically using ε302 = 1670 m ^−1^ cm^−1^ before each experiment. Sodium hydroxide (0.3 m NaOH) was used as a vehicle control, as peroxynitrite is supplied in NaOH to maintain its stability. Nitration of peptides was measured under basic conditions by raising the pH of NaOH to better distinguish the 3‐nitrotyrosine peaks. 3‐Nitrotyrosine has a characteristic spectral shift upon alkanization, which is reflected by the secondary maximum shifting from 357 to 430 nm.^68^, ^69^

*Peptide‐Conjugated Fluorescent Particles*: The peptides were covalently bound to the carboxyl‐functionalized fluorescent particles using standard EDC/NHS chemistry, as illustrated in Figure 1. The fluorescent particles were initially washed with 0.1 m MES buffer (pH 4.7), followed by activation of surface carboxylic acids with EDC (≈20 × 10^−3^ m) and NHS (≈50 × 10^−3^ m) for 15 min before conjugation in MES buffer. Once the carboxyl groups were activated, the pH of the buffer was then raised from 4.7 to 7.4 using PBS to improve coupling efficiency. Tyrosine‐containing peptides (P1–P4) were then conjugated to the FPs; each peptide was conjugated to the FPs at 1 × 10^−3^ m final concentration in PBS and incubated overnight on a temperature‐controlled shaker at 1000 rpm and 4 °C (Thermomixer, Eppendorf). After incubation, the peptide–FP complex was washed with PBS three times; the unbound peptides were removed after each centrifugation step (10 min at 14 000 rpm). Finally, the peptide–FP complex was re‐suspended in PBS at 1% (v/v) stock solution.

*3‐Nitrotyrosine Detection on Fluorescent Particle Complex*: To induce nitration, each peptide–FP complex was incubated with varying concentrations of peroxynitrite in a 96‐well plate and incubated for 1 h at 37 °C, followed by 3‐nitrotyrosine detection. Fluorescent particles conjugated with 3‐nitrotyrosine amino acids served as a positive control for all immunoassay studies while NaOH (0.3 m)‐treated peptide–FP complexes were used as negative vehicle controls. To determine the sensitivity of 3‐nitrotyrosine conversion, peptide–FP complexes were loaded on a nitrocellulose membrane with a Bio‐Dot microfiltration system using gravity flow (Bio‐Rad Laboratories, Inc.). After loading, the membranes were incubated in 5% nonfat dry milk in Tris‐buffered saline containing 0.2% Tween‐20 (TBS‐T), which served as a blocking buffer. Nitrated FPs were detected with a mouse monoclonal anti‐nitrotyrosine antibody (1:500) diluted in blocking buffer and incubated with membranes overnight at 4 °C. Membranes were then washed with TBS‐T (four times, 10 min each) and incubated for 1 h at room temperature in a goat anti‐mouse IgG secondary antibody (1:5000) diluted in blocking buffer. This was followed by TBS‐T wash (four times, 10 min each) prior to visualization. Immunoreactivity was visualized using an infrared imaging system (Odyssey CLx, LI‐COR), where the 3‐nitrotyrosine signals were detected at 778_ex_/794_em_, whereas the FPs were detected at 580_ex_/605_em_. 3‐Nitrotyrosine antibody signal (*G*_i_) was corrected for background binding signal (*G*_b_) obtained from the vehicle‐treated peptide–FPs and then divided by the particle's fluorescence of each peptide–FP complex (*R*_i_), allowing for normalization of 3‐nitrotyrosine signal to concentration of FPs loaded on the nitrocellulose membrane. The background fluorescence from the fluorescent particles was negligible and thus was not included in the correction for the fluorescence signals. Each experimental reading was carried out in triplicates to minimize dot‐to‐dot variations in loading; thus, a normalized 3‐nitrotyrosine antibody signal (G*) was obtained as represented in Equation (1)

(1) $$G*\;\;=\;\;\frac{G_{i}-G_{b}}{R_{i}}$$

*Cell Culture*: HUVEC‐2 (BD Biosciences, MA) were cultured in Medium 199 (Invitrogen) supplemented with 15% fetal bovine serum (Premium select; Atlanta Biologics), heparin sodium salt (90 µg mL^−1^), endothelial mitogen (0.1 mg mL^−1^; Biomedical Technologies, MA), penicillin (100 U mL^−1^), streptomycin (0.1 mg mL^−1^), and glutamine (0.29 mg mL^−1^) at 37 °C and 5% CO_2_. Cells from passages 3–7 were used for the shear stress experiments.

*Shear Stress Experiments*: HUVECs were seeded at confluence (1 × 10^5^ cells cm^−2^) into µ‐slides I^0.6^ (Ibidi, Munich, Germany) and cultured overnight (≈18–20 h) in an incubator at 37 °C with 5% CO_2_ to allow for cell attachment. The µ‐slides were then connected to the Ibidi pump system (Ibidi) and exposed to either low (1.5 dynes cm^−2^) or high (15 dynes cm^−2^) shear stress for 24 h. Cells exposed to the high shear were subjected to a gradual increase in shear stress levels over the first hour (1.5, 5–15 dynes cm^−2^) to allow for adaptation to continuous laminar flow (following the manufacturer's protocol). Each µ‐slide was loaded with 0.01% (v/v) of peptide–FP complexes in the media at the start of the shear experiment. After 24 h of shear stress, the medium was collected and the peptide–FPs were pelleted (30 min at 14 000 rpm). The supernatant was frozen at −20 °C and used for quantifying nitrite concentration using the Griess assay. The peptide–FPs (in the pellet) were washed and centrifuged in PBS for 10 min at 14 000 rpm; this process was repeated three times. The peptide–FPs were resuspended in PBS for immunodetection of 3‐nitrotyrosine by dot blot.

*Immunofluorescence Microscopy*: Following each shear stress experiment, cells were fixed with 4% paraformaldehyde in PBS at 4 °C for 30 min. Cells were washed in PBS (three times, 5 min each) then permeabilized with 0.2% Triton X‐100 in PBS for 15 min at room temperature and blocked with 10% goat serum in PBS for 1 h or overnight at 4 °C. The cells were then incubated with an antibody raised in rabbit against VE‐cadherin at a dilution of 1:400 in blocking solution for 3 h at room temperature, followed by Alexa Fluor 488 goat anti‐rabbit secondary antibody diluted 1:1000 in PBS for 1 h. Finally, nuclei were labeled by incubating the cells for 5 min at room temperature in 2 µg mL^−1^ 4′,6‐diamidino‐2‐phenylindole (DAPI) in PBS and mounted in ProLong Diamond antifade reagent. Images were acquired using a Leica SP5 confocal microscope with a 20× objective.

*Statistical Analysis*: To analyze the data, a two‐tailed Student *t*‐test was performed to determine the statistical differences between treatment groups. The statistical significance threshold was taken to be a *p*‐value of 0.05. Results are expressed as means ± standard deviations (SD). EC_50_ values were calculated based on the normalized dose–response curves and were analyzed by GraphPad 7.0 (Prism, La Jolla, CA, USA).

## Conflict of Interest

JYHC, DRO, LWC and WDS are named inventors on a pending patent covering the technology described within the manuscript.

## Supporting information

Supplementary
