# Supplementary material for: Peptide‐Functionalized Fluorescent Particles for In Situ Detection of Nitric Oxide via Peroxynitrite‐Mediated Nitration
Source: Adv Healthc Mater. 2017 May 17;6(16):1700383. doi: 10.1002/adhm.201700383 (PMC5568941; doi:10.1002/adhm.201700383)
Supplement: Supplementary file 1 — Supplementary [file ADHM-6-na-s001.pdf]

Copyright WILEY-VCH Verlag GmbH & Co. KGaA, 69469 Weinheim, Germany, 2017.

ADVANCED HEALTHCARE MATERIALS

Supporting Information

for *Adv. Healthcare Mater.*, DOI: 10.1002/adhm.201700383

Peptide-Functionalized Fluorescent Particles for In Situ Detection of Nitric Oxide via Peroxynitrite-Mediated Nitration

Jason Y. H. Chang, Lesley W. Chow, W. Michael Dismuke, C. Ross Ethier, Molly M. Stevens, W. Daniel Stamer, and Darryl R. Overby**

Copyright WILEY-VCH Verlag GmbH & Co. KGaA, 69469 Weinheim, Germany, 2016.

Supporting Information

Jason Y.H. Chang, Lesley W. Chow, W. Michael Dismuke, C. Ross Ethier, Molly M. Stevens, W. Daniel Stamer, and Darryl R. Overby**

Dr. J. Y. H. Chang,[+] Prof. C. R. Ethier, Dr. D. R. Overby
Department of Bioengineering
Imperial College London
London SW7 2AZ, UK
E-mail: d.overby@imperial.ac.uk

Dr. L.W. Chow,[++] Prof. M. M. Stevens
Department of Materials, Department of Bioengineering, and Institute of Biomedical Engineering
Imperial College London
London SW7 2AZ, UK

Prof. C. R. Ethier
Wallace H. Coulter Department of Biomedical Engineering
Georgia Institute of Technology and Emory University
Atlanta, GA 30332, USA

Dr. J.Y.H. Chang, Dr. W. M. Dismuke, Prof. W. D. Stamer
Department of Ophthalmology
Duke University School of Medicine
Durham, NC 27710, USA
E-mail: dan.stamer@duke.edu

[+] Present address: Koch Institute for Integrative Cancer Research, Massachusetts Institute of Technology, 77 Massachusetts Ave, Cambridge, MA 02139, USA

[++] Present address: Department of Materials Science and Engineering and Bioengineering Program, Lehigh University, Bethlehem, PA 18015, USA

Table S1: Comparison of NO detection platforms.

	Principle of Detection	*Detection Limit*	*Spatial Localization*	*Comments*
**Griess Assay**	NO_2-/NO_3- sensitive dye that detects the portion of NO that is oxidized to NO_2-/NO_3-	**0.5-1 μM**, cumulative measurement, as NO_2-/NO_3- accumulates over time	Localization not possible because measures NO_2-/NO_3- in bulk solution	•Gold standard for measuring NO in biological systems
**Fluorescent Methods**	NO-sensitive fluorophore that can be used in solution or trapped within individual cells	**3-5 nM** in cell-free systems, but in biological samples detection is degraded to μM levels[a–d]. Cumulative measurement, as fluoro-phores bind irreversibly to NO	Localization possible, but requires optical access	•Sensors may photo-bleach<br>•Sensors cross-react with other oxygen/nitrogen species present biological samples
**Electrochemical**	Electrochemical probe sensitive to several reactive oxygen/nitrogen species covered by NO-selective membrane	**~ 10 nM**, instantaneous measurement proportional to NO concentration at probe tip	Localization possible, but requires probe tip to be implanted within tissue	•Invasive probe may disrupt tissue architecture and function<br>•Delicate probe tip
**Peptide-functionalized Particles (our method)**	Fluorescent particles coated with nitration-sensitive peptides detected afterwards using 3-nitrotyrosine antibodies	**~100 nM**, cumulative measurement, as 3-nitrotyro-sine accumulates over time	Localization possible, but confined to the bead surface	•Localisation requires histology<br>•Measures NO via ONOO-medi-ated peptide nitration<br>•Detection limit that is more sensitive than Griess or DAF-FM in biological samples

[a] H. Kojima, N. Nakatsubo, K. Kikuchi, S. Kawahara, Y. Kirino, H. Nagoshi, Y. Hirata, T. Nagano, Anal. Chem. 1998, 70, 2446.
[b] Z. Lacza, E. M. Horváth, E. Pankotai, A. Csordás, M. Kollai, C. Szabó, D. W. Busija, J. Pharmacol. Toxicol. Methods 2005, 52, 335.
[c] P. Lorenz, S. Roychowdhury, M. Engelmann, G. Wolf, T. F. W. Horn, Nitric Oxide - Biol. Chem. 2003, 9, 64.
[d] S. Roychowdhury, A. Luthe, G. Keilhoff, G. Wolf, T. F. W. Horn, Glia 2002, 38, 103.

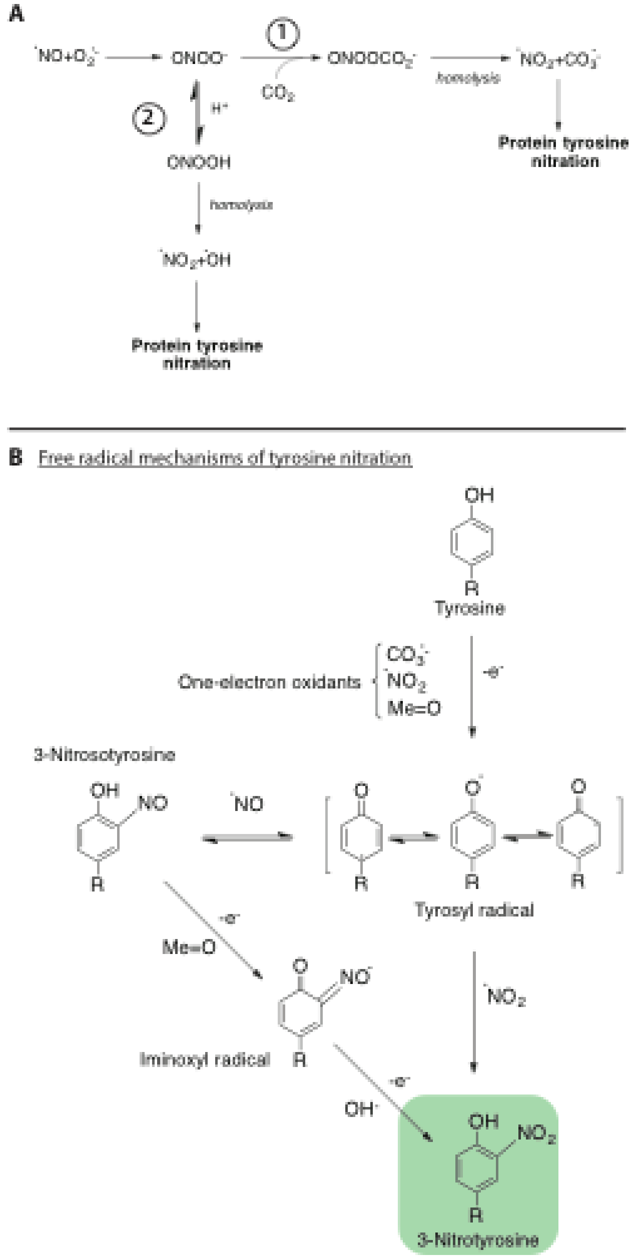

Figure S1: Biochemical pathway of peroxynitrite formation and tyrosine nitration. **(A)** Formation of peroxynitrite anion (ONOO–) through the diffusion-limited reaction of NO and $O_2^{\bullet}$–) radicals. **(1)** A fundamental reaction of ONOO– in biological systems is with carbon dioxide (CO2), which results in an intermediate adduct, nitroso-peroxocarboxylate ($ONOOCO_2$—) that leads to the formation of carbonate (CO_3—) and nitrogen dioxide ($^{\bullet}NO_2$) radicals. **(2)** Alternatively, ONOOH can undergo homolytic fission to generate hydroxl ($^{\bullet}$OH) and $^{\bullet}NO_2$ radicals.[47] **(B)** These one-electron oxidants promote the formation of tyrosyl radicals ($^{\bullet}$Tyr) which then combines with $^{\bullet}NO_2$ to form 3-nitrotyrosine. *Schematic diagram is adapted from Radi et al.*[41,44,50]

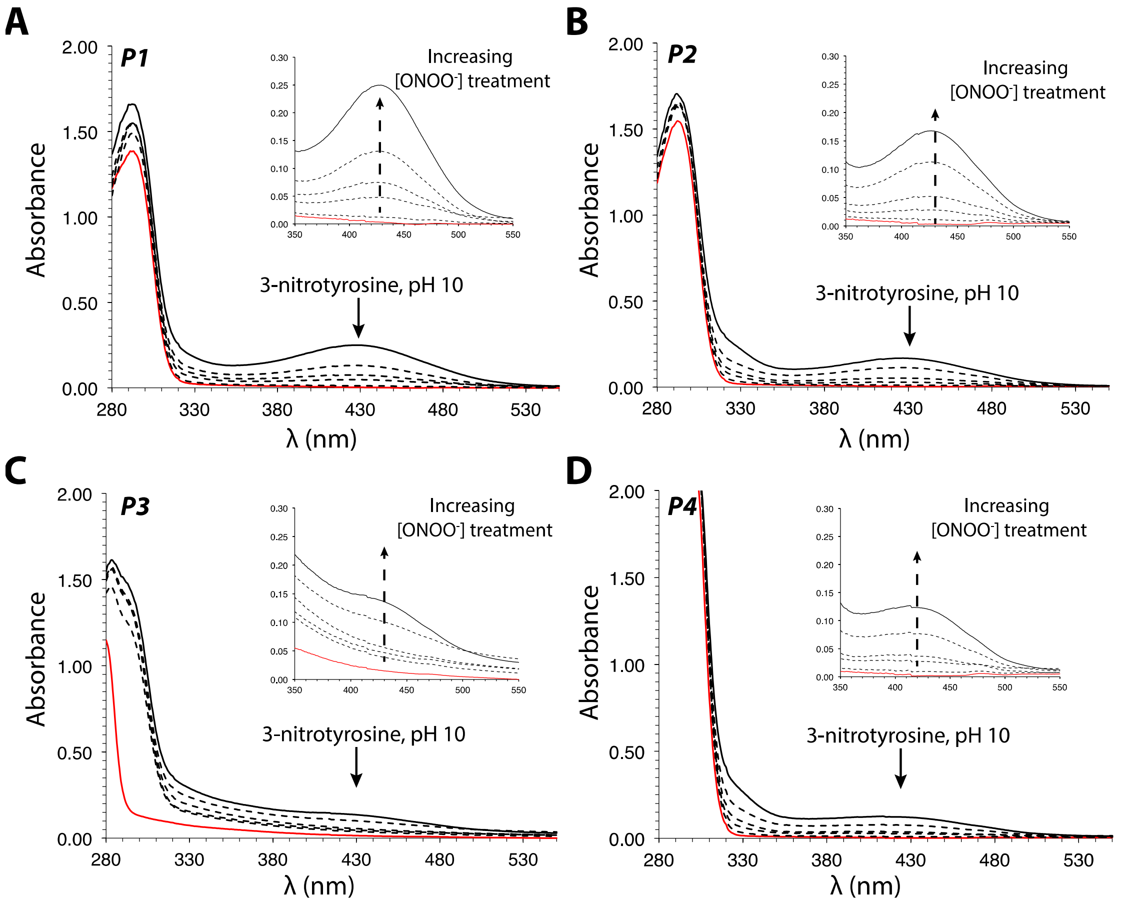

Figure S2: Nitration sensitivity of synthetic tyrosine-containing peptides measured by UV-VIS spectrophotometry. **(A-D)** Nitration sensitivity of peptides (P1-4) as a function of increasing concentration of peroxynitrite (10 µM, 50 µM, 100 µM, 250 µM and 500 µM; black lines). 3-Nitrotyrosine absorbance is measured at 430nm under basic conditions (pH ~ 10). (*Inset*) Magnification of absorbance values at 430nm for each nitrated peptide as a function of peroxynitrite treatment. *Red lines* = vehicle treated controls (0.3M NaOH).

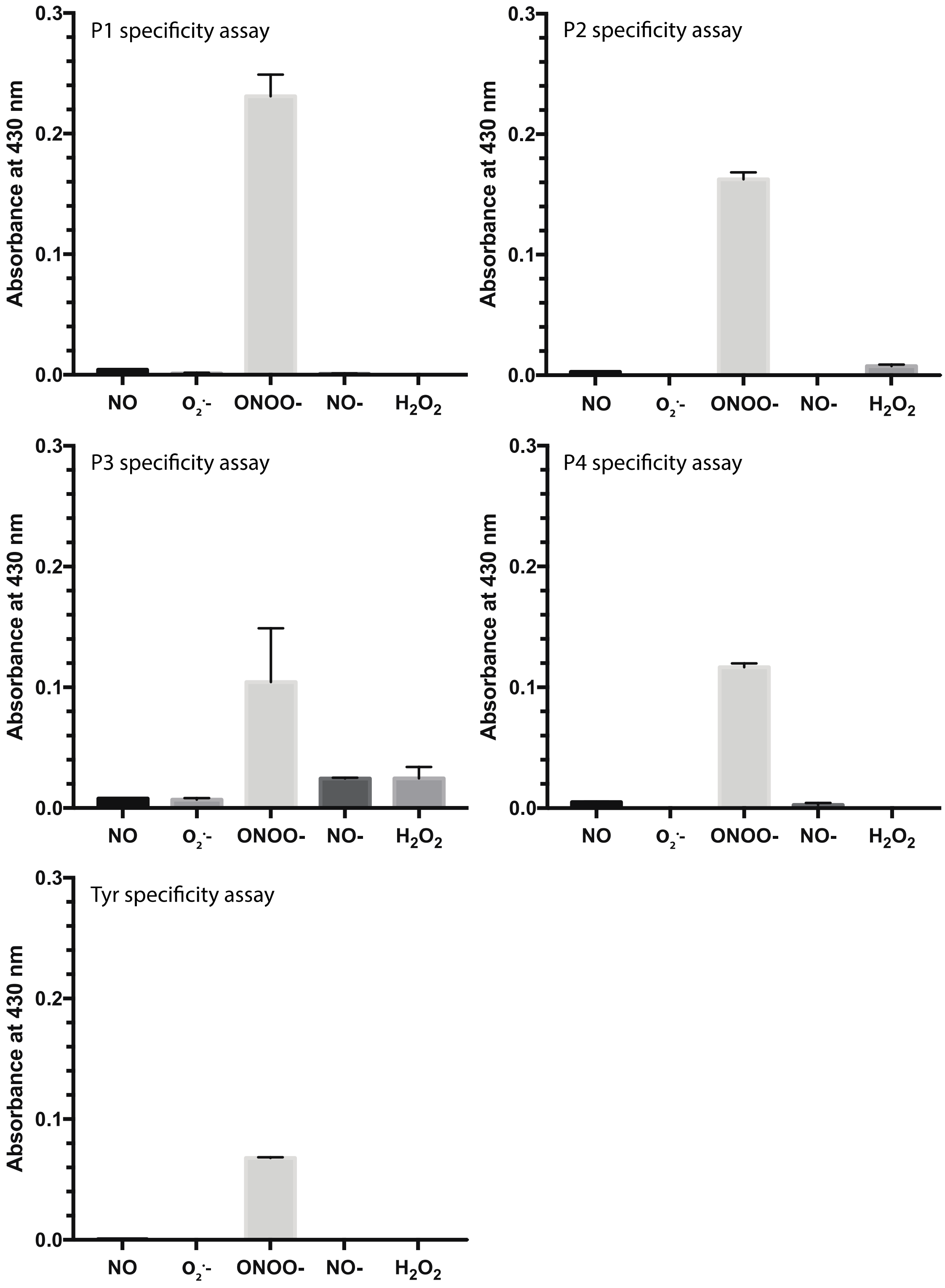

Figure S3: Peptide specificity assay treated with various reactive oxygen and nitrogen species (N=3). Nitric oxide: NO, superoxide: $^{\bullet}O_2$ –, peroxynitrite: ONOO–, Angeli's salt: NO– and hydrogen peroxide: H_2O_2 . Superoxide is generated by xanthine/xanthine oxidase system.[70]

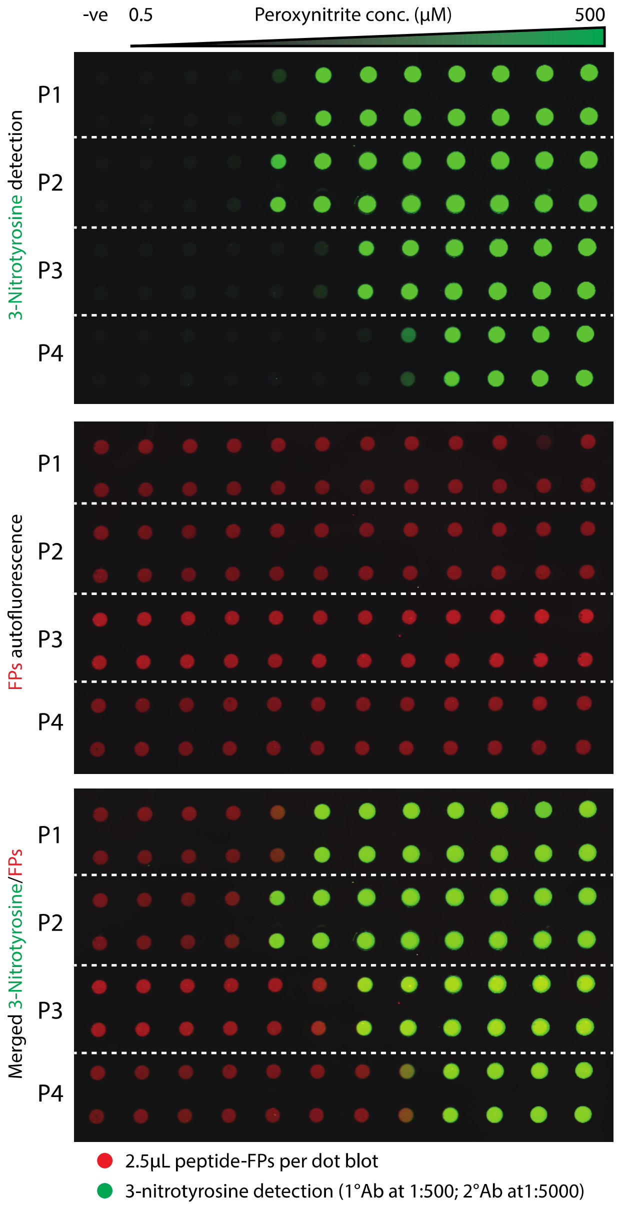

Figure S4: Representative peptide-conjugated fluorescent particle complex dot blot sensitivity immunoassay. **(Top)** 3-Nitrotyrosine detection for each of the synthetic peptide-conjugated fluorescent particle complexes with varying concentration of peroxynitrite treatment (green). **(Middle)** Fluorescent particles to quantify the loading concentration per sample (red). **(Bottom)** Overlay of 3-nitrotyrosine and fluorescent particles. -*ve* = Vehicle treated controls (0.3M NaOH).

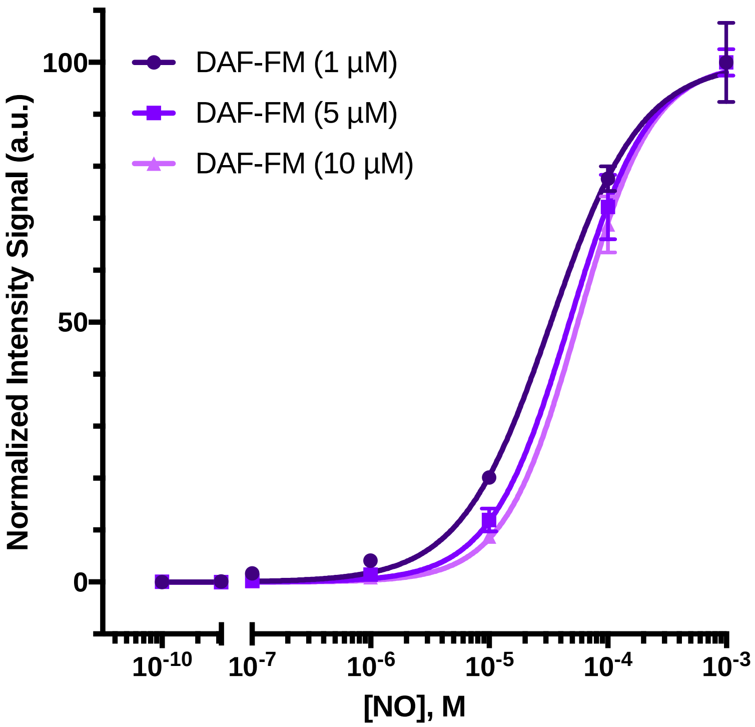

Figure S5: Detection of NO with varying concentrations of DAF-FM probe.

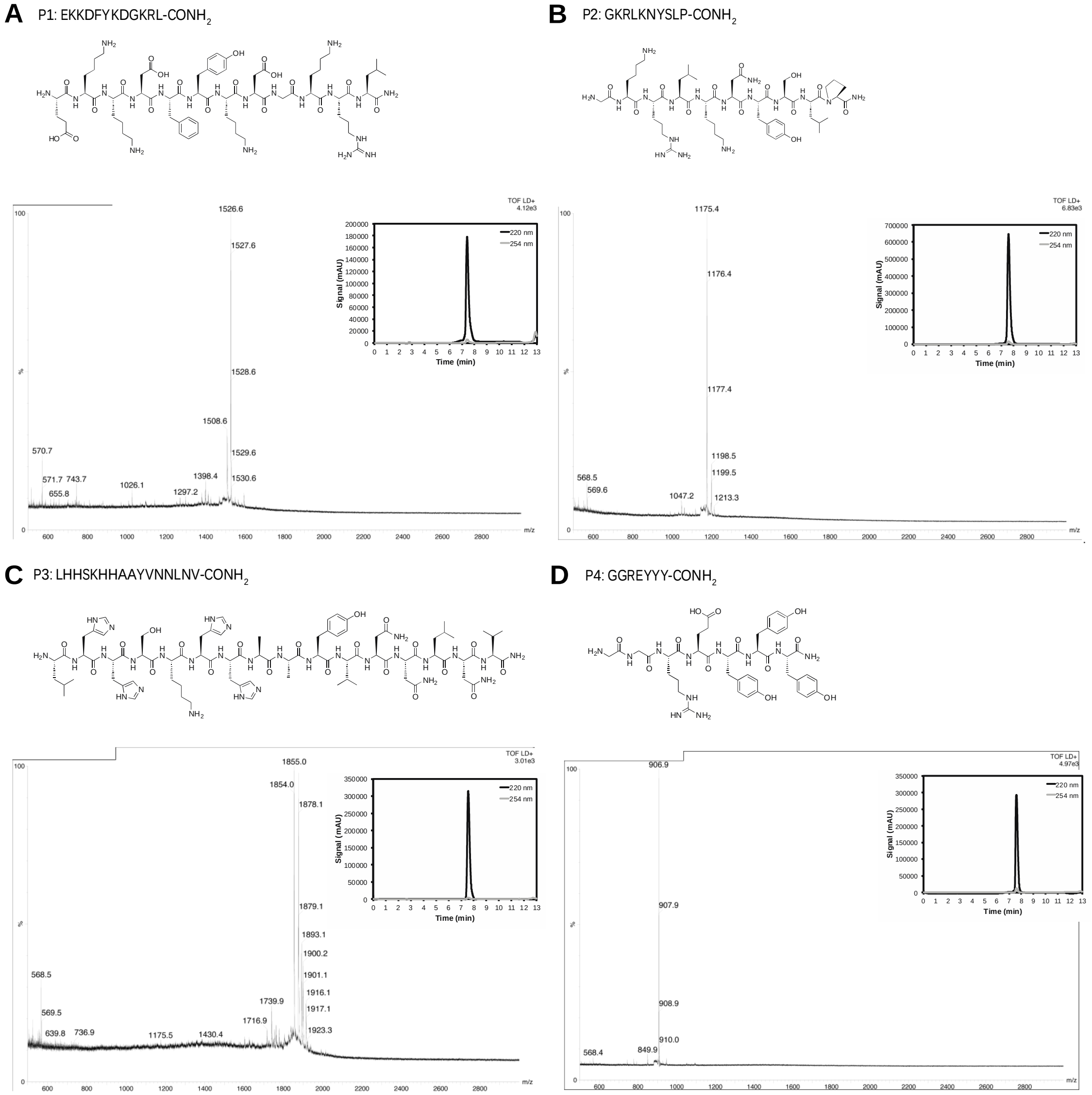

Figure S6: Chemical structures, MALDI and analytical HPLC trace of the purified peptide sensors. (A) P1: EKKDFYKDGKRL (MW 1526). (B) P2: GKRLKNYSLP (MW 1175). (C) P3: LHHSKHHAAYVNNLNV (MW 1854). (D) P4: GGREYYY (MW 906).
